# Evidence of Oxidative Stress and Secondary Mitochondrial Dysfunction in Metabolic and Non-Metabolic Disorders

**DOI:** 10.3390/jcm6070071

**Published:** 2017-07-19

**Authors:** Karolina M. Stepien, Robert Heaton, Scott Rankin, Alex Murphy, James Bentley, Darren Sexton, Iain P. Hargreaves

**Affiliations:** 1 The Mark Holland Metabolic Unit Salford Royal NHS Foundation Trust Stott Lane, Salford M6 8HD, UK; 2 School of Pharmacy, Liverpool John Moore University, Byrom Street, Liverpool L3 3AF, UK; r.heaton@2013.ljmu.ac.uk (R.H.); s.rankin@2014.ljmu.ac.uk (S.R.); a.murphy3@2013.ljmu.ac.uk (A.M.); j.bentley@2014.ljmu.ac.uk (J.B.); d.w.sexton@ljmu.ac.uk (D.S.)

**Keywords:** mitochondria, electron transport chain, reactive oxygen species, reactive nitrogen species, oxidative stress, phenylketonuria, methylmalonic acidemia, methylmalonic acid, peroxisome, glutathione, catalase, superoxide dismutase, coenzyme Q_10_, sepsis, nitrosative stress, nitric oxide synthase

## Abstract

Mitochondrial dysfunction and oxidative stress have been implicated in the pathogenesis of a number of diseases and conditions. Oxidative stress occurs once the antioxidant defenses of the body become overwhelmed and are no longer able to detoxify reactive oxygen species (ROS). The ROS can then go unchallenged and are able to cause oxidative damage to cellular lipids, DNA and proteins, which will eventually result in cellular and organ dysfunction. Although not always the primary cause of disease, mitochondrial dysfunction as a secondary consequence disease of pathophysiology can result in increased ROS generation together with an impairment in cellular energy status. Mitochondrial dysfunction may result from either free radical-induced oxidative damage or direct impairment by the toxic metabolites which accumulate in certain metabolic diseases. In view of the importance of cellular antioxidant status, a number of therapeutic strategies have been employed in disorders associated with oxidative stress with a view to neutralising the ROS and reactive nitrogen species implicated in disease pathophysiology. Although successful in some cases, these adjunct therapies have yet to be incorporated into the clinical management of patients. The purpose of this review is to highlight the emerging evidence of oxidative stress, secondary mitochondrial dysfunction and antioxidant treatment efficacy in metabolic and non-metabolic diseases in which there is a current interest in these parameters.

## 1. Introduction

Oxidative stress has been implicated as a major contributory factor to the pathophysiology of a number of diseases and conditions including cancer [1], sepsis [2] and metabolic diseases [3,4,5,6,7,8]. The origin of oxidative stress in disease is generally multifactorial and can rarely be attributed to one mechanism [9]. Although, impairment of mitochondrial function as a secondary consequence of disease pathophysiology is thought to make a major contribution to reactive oxygen species (ROS) generation in a number of disorders [9]. Factors responsible for this mitochondrial dysfunction include toxic metabolites which accumulate in metabolic disorders [10,11] as well as ROS and reactive nitrogen species (RNS) generated as part of the pathogenesis of other diseases [2,12]. These factors are then able to directly impair the electron transport chain (ETC) which is the site of mitochondrial ROS generation [13,14].

The cell has several means available to tackle free radical generation including antioxidants and antioxidant enzymes; however, as soon as pro-oxidants exceed the antioxidant capacity of the cell, free radicals accumulate and oxidative stress occurs with the resultant damage to proteins, lipids and DNA causing cellular and consequently organ dysfunction [9]. In view of the detrimental effects of oxidative stress, a number of studies have investigated the utility of antioxidant interventions in disease and have shown evidence of therapeutic efficacy in some cases [15,16].

It is the purpose of this review to highlight evidence of oxidative stress and secondary mitochondrial dysfunction in disease, highlighting putative mechanisms and therapeutic strategies in disorders in which there is a growing interest in the association between these parameters. Although this review will primarily focus upon oxidative stress, evidence of nitrosative stress as the result of RNS accumulation will also be outlined in the metabolic and non-metabolic diseases discussed in this review.

## 2. Phenyloketonuria (PKU)

PKU is an autosomal recessive inherited metabolic disorder of amino acid metabolism which is caused by mutations in the gene encoding the enzyme, phenylalanine hydroxylase (EC1.14.16.1) [17]. Phenylalanine (Phe) is an essential amino acid obtained exclusively from the diet or by proteolysis. It is crucial for protein synthesis, as well as for the synthesis of tyrosine and its derivatives, such as dopamine, norepinephrine and melanin [18,19]. However, a deficiency of phenylalanine hydroxylase leads to accumulation of Phe in the blood and other tissues of affected patients [20,21,22]. Phe concentrations in plasma may reach very high levels (mmol/L) and, as a result, some of the accumulated Phe can then be metabolized by alternative pathways making phenylketones such as phenylpyruvate, phenyllactate and phenylacetate [20].

Untreated PKU patients present with severe mental retardation, microcephaly, developmental delay, epilepsy, behavioral alterations, cerebral white matter abnormalities and progressive supranuclear motor disturbances [17,23,24]. Newborn screening for PKU has enabled early diagnosis and treatment of this condition [25]. This will help prevent the possibility of mental retardation, although slightly reduced neurophysiological outcomes may occur, in particular in combination with poor compliance to PKU diet [26]. The main findings presented by PKU patients are severe neurological damage, including corpus callosum, striatum, and cortical alterations and hypomyelination, that result in intellectual deficit and neurodegeneration [27,28,29,30]. However, the pathophysiology underlying the brain damage has yet to be fully elucidated, although oxidative stress may play an important role [15]. In PKU, oxidative stress appears to be already present at the time of diagnosis and persists even in the presence of dietary compliance [31,32]. Evidence of oxidative stress in PKU patients has been indicated by increased levels of plasma thiobarbituric acid-reactive species (TBAR), an indicator of lipid peroxidation [33], malondialdehyde (a lipid peroxidation marker) [31] and 8-hydroxy-2-deoxygyanosine (marker of DNA oxidation) [34]. The oxidative stress associated with PKU may result from the effect of the restricted diet of patients as well as the elevated levels of Phe or its metabolites upon cellular antioxidant defenses [15]. Historically, a deficiency in the status of the trace metal, selenium (Se), was considered to be an important contributory factor to the oxidative stress associated with PKU [35]. Se is required for the biological activity of selenoproteins, one of which is the antioxidant enzyme, glutathione peroxidase (GSH-Px; EC: 1.11.1.9), and therefore, a deficiency in Se status may compromise the activity of this enzyme [36]. However, evidence of decreased GSH-Px activity has been reported in PKU patients with plasma Se levels within the reference range suggesting that other factors may be responsible for the deficit in enzyme activity [33]. One of these factors may be the low level of methionine present in the diet of PKU patients, which may result in impaired GSH-Px synthesis [5]. Phe itself may directly inhibit the activity of GSH-Px [33]. In addition, animal studies have reported the potential for hyperphenylalaninemia to directly suppress the production of GSH-Px as well as enhance its degradation [37]. A decreased level of the cellular antioxidant, reduced glutathione (GSH), has also been reported in PKU, although it was uncertain whether this was caused by oxidative stress or the restricted diet [38]. However, a subsequent study in rat astrocytoma cells reported evidence of decreased GSH status in conjunction with increased oxidative stress in cells exposed to Phe at levels commonly detected in PKU patients (1000–1500 μmol/L) [39]. This study indicated the vulnerability of neural cells to Phe-induced oxidative stress which may be an important contributory factor to the neurological dysfunction associated with PKU. Kienzle-Hagen and colleagues (2002) reported a significant (*p* < 0.01) inhibitory effect of the hyperphenylalaninemia on the cerebral catalase activity of rat [37]; however, studies in PKU patients have found no evidence of an inhibition of this enzyme in peripheral tissue [31]. Indeed, a number of studies have reported an increase in the activity of this enzyme in patients [40]. 

In addition to oxidative stress, one study has reported evidence of nitrosative stress in PKU patients by measurement of serum NO*x* (nitrite/nitrate), the stable breakdown products of nitric oxide (NO), which was found to be significantly increased compared to control levels [33]. However, NO*x* tended to be lower in patients with plasma Phe levels > 900 μM. This study suggested an impairment in the regulation of NO metabolism in PKU with the increase in serum NO*x* < 900 μM Phe thought to reflect the increased oxidative stress. The decrease in serum NO*x* at Phe > 900 μM originates from the oxidative stress-induced transcriptional suppression of the nitric oxide synthase (NOS) gene, or as a result of structural changes in the NOS enzyme [33].

The mevalonate pathway enzymes, 3-hydroxy-3-methylglutaryl-CoA (HMG-CoA; EC1.1.1.98) reductase, and mevalonate 5-pyrophosphate decarboxylase (EC4.1.1.33) have been reported to be inhibited by Phe and its metabolite, phenylacetate; however, only Phe-induced inhibition within its physiological range (≥250 μmol/L) [41]. Since HMG-CoA reductase is the major regulatory enzyme in the synthesis of both cholesterol and the lipid soluble antioxidant, coenzyme Q_10_ (CoQ_10_), since they share a common pathway, it is therefore unsurprising that perturbations in the synthesis of both of these isoprenoids have been associated with PKU [6,42]. The availability of tyrosine is also essential for the synthesis of CoQ_10_; however, in PKU, no association has been observed between the plasma level of tyrosine and that of CoQ_10_, although this relationship was not investigated in tissues [6]. The results of cellular CoQ_10_ status in PKU has been contradictory with a study by Colome et al. (2002) finding evidence of a deficit in this isoprenoid in the lymphocytes from well-controlled PKU patients [43]. In contrast, a study by Hargreaves et al. (2002) found no evidence of a CoQ_10_ deficiency in blood mononuclear cells from an older group of PKU patients [44].

The reported ability of hyperphenylalaninaemia to impair the activity of the mitochondrial electron transport chain (ETC) [45] may also contribute to the oxidative stress associated with PKU, since ETC dysfunction has been associated with reactive oxygen species (ROS) generation [13]. In the study by Rech et al. (2002), ETC complex I–III (NADH cytochrome c reductase; EC1.3.5.1 + EC1.10.2.2) activity was found to be reduced following chemically induced hyperphenylalaninemia in rat brain cortex [45]. ETC complex II (succinate: ubiquinone reductase; EC1.3.5.1) and complex IV (cytochrome c oxidase; EC1.9.3.1) were unaffected. It was surmised that the impairment of ETC complex I–III activity was the result of Phe competing with NADH for the active site of complex I (NADH ubiquinone reductase; EC: 1.6.5.3). Subsequent studies in human astrocytoma cells [46] and blood mononuclear cells [44] have found no evidence of inhibition of either ETC complex I or ETC complex II–III (succinate:cytochrome reductase; EC1.3.5.1 + EC1.10.2.2) activities, respectively under conditions of hyperphenylalaninemia. However, since no studies have as yet directly assessed the effect of hyperphenylalaninemia on ETC complex III (ubiquinol: cytochrome c reductase; EC1.10.2.2) activity, the possibility that this enzyme is susceptible to Phe-induced toxicity cannot be discounted. In addition, the suggested ability of hyperphenylalaninemia to induce a CoQ_10_ deficiency in some studies may also result in secondary ETC dysfunction in some PKU patients [6,43].

**Figure 1:**
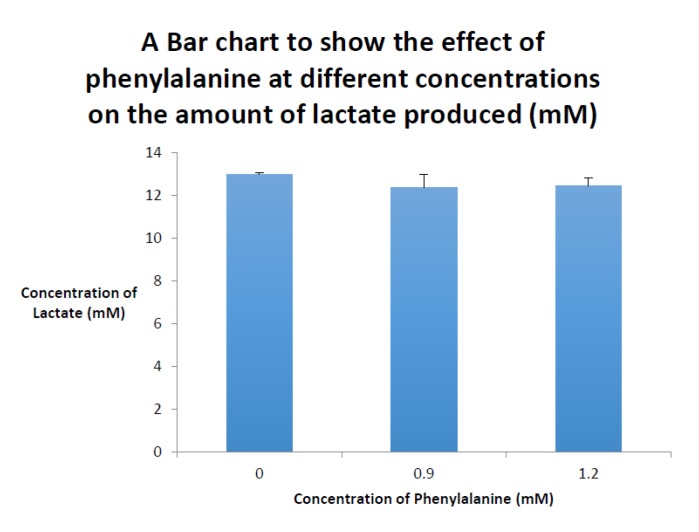
Bar chart displaying the mean cell culture lactate concentrations determined following culture of human HEPG2 liver cells for 72 h with 0.9 and 1.2 mM phenylalanine, respectively. Results are expressed as the mean and standard deviation of four determinations.

The effect of hyperphenylalaninaemia on the mitochondrial oxidative metabolism was investigated by the authors by determining the lactate concentration of cell culture medium derived from immortalised HEPG2 liver cells that had been exposed to 900 and 1200 μmol/L Phe, respectively, for 72 h. Following 72 h of culture, the lactate concentration in the cell culture media was determined by the method outlined in the study by Kyprianou et al. (2009) and no significant difference was found between the control and Phe-treated HEPG2 cell groups following Student’s *t*-test analysis (*p* < 0.05 was considered statistically significant, Figure 1) [46], which suggests no evidence of Phe-induced ETC impairment in the immortalised human liver cells.

**Figure 2:**
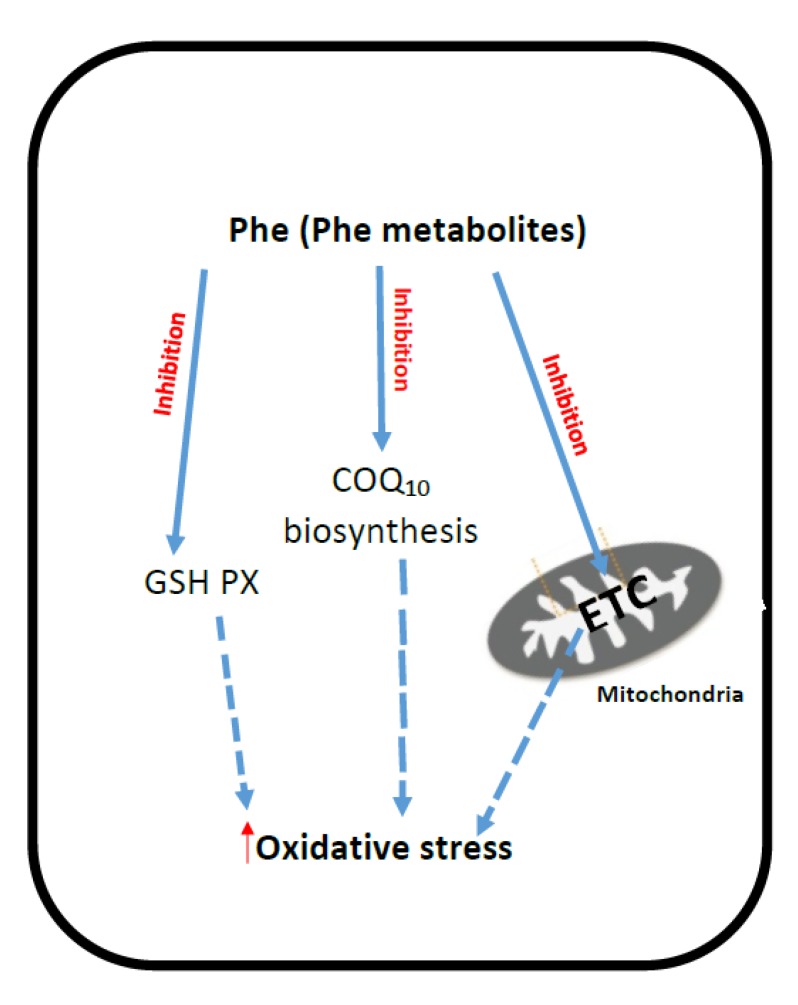
Putative mechanisms of oxidative stress generation and mitochondrial dysfunction in PKU. PKU: Phenylketonuria; Phe: Phenylalanine; ETC: Mitochondrial electron transport chain; CoQ_10_: Coenzyme Q_10_; GSH-PX: Glutathione peroxidase.

The putative mechanisms that have been implicated for ETC dysfunction and oxidative stress in PKU are outlined in Figure 2.

Treatments for PKU patients consist of restriction of Phe intake, through natural-protein-restricted diet supplemented with Phe-free amino acid mixtures enriched with trace elements, vitamins and minerals [47,48,49]. Strict low-protein diet, however, causes some micronutrient and antioxidant deficiencies including zinc, copper, Se, magnesium and iron (Fe) deficiencies [50,51,52,53]. A deficiency in Fe may also result in a secondary diminution in the level of carnitine, since Fe is required for the synthesis of this compound [54]. In view of the antioxidant properties of carnitine, which is able to act as an ROS scavenger, a deficit in the status of this compound which has been reported in some PKU patients may comprise antioxidant status [38,55]. Indeed, supplementation of PKU patients with Se and carnitine has been recommended as a means to ameliorate the oxidative stress associated with this condition [35]. At present however, there is no overall consensus on the use of antioxidant supplementation in the treatment of PKU, although this adjunct therapy may offer some protection against the neurological dysfunction associated with this condition [56].

## 3. Methylmalonic Acidemia

Methylmalonic acidemia is one of the organic acidemias, which is primarily caused by severe deficiency of the enzyme, l-methylmalonyl-CoA mutase (MCM; EC: 5.4.99.2), or by defects in the synthesis of 5-deoxyadenosyl cobalamin, the active form of vitamin B12 and an essential cofactor required for the activation of MCM [57]. This condition leads to an increase in the level of methylmalonyl-CoA, which is spontaneously converted to methylmalonic acid (MMA) [58]. Biochemically, the condition is characterized by tissue accumulation of MMA. The levels of MMA in the blood and cerebrospinal fluid are usually around 2.5 mmol/L during acute metabolic crises [58,59] but may be even higher in the brain [60].

Clinical features of this condition include lethargy, coma, vomiting, failure to thrive, muscular hypotonia, progressive neurological deterioration and kidney failure [61].

The mechanisms responsible for the neurological and renal dysfunction in this organic acidemia have so far not been fully elucidated, although ETC dysfunction and oxidative stress are thought to contribute to the pathophysiology of this disorder [62,63].

Evidence of ETC dysfunction in methylmalonic acidemia was first suggested by the unexplained lactic acidosis in patients with this condition [64]. This was later confirmed in the study Hayasaka et al. (1982), which reported evidence of ETC complex IV deficiency in post-mortem liver of a single patient [62]. A number of subsequent studies have demonstrated evidence of ETC dysfunction in association with methylmalonic acidemia, with evidence of both single [65,66] and multiple ETC enzyme deficiencies [67,68,69,70] being reported in patient and animal studies. In addition, animal and patient studies have also reported morphological abnormalities in mitochondria as the result of methylmalonic acidemia. Proteinuria, renal tubular injury, dilated tubuli and mitochondrial swelling and disorganization of cristae in the tubulum epithelium was observed in an experimental study on rats exposed chronically to MMA [71]. Cell autonomous ETC complex IV deficiency was demonstrated in megamitochondria from renal tubules in a patient with MMA [72], confirming the observations from the previous animal study [71]. Brain imaging and histopathological investigations have revealed a symmetric degeneration of the basal ganglia, particularly the globus pallidus, as well as a mild spongiosis of the subthalamic nucleus, mammillary bodies, and internal capsule [73,74,75]. Interestingly, symmetrical lesions in the basal ganglia are also found in patients with inherited ETC complex II deficiencies [76].

**Figure 3:**
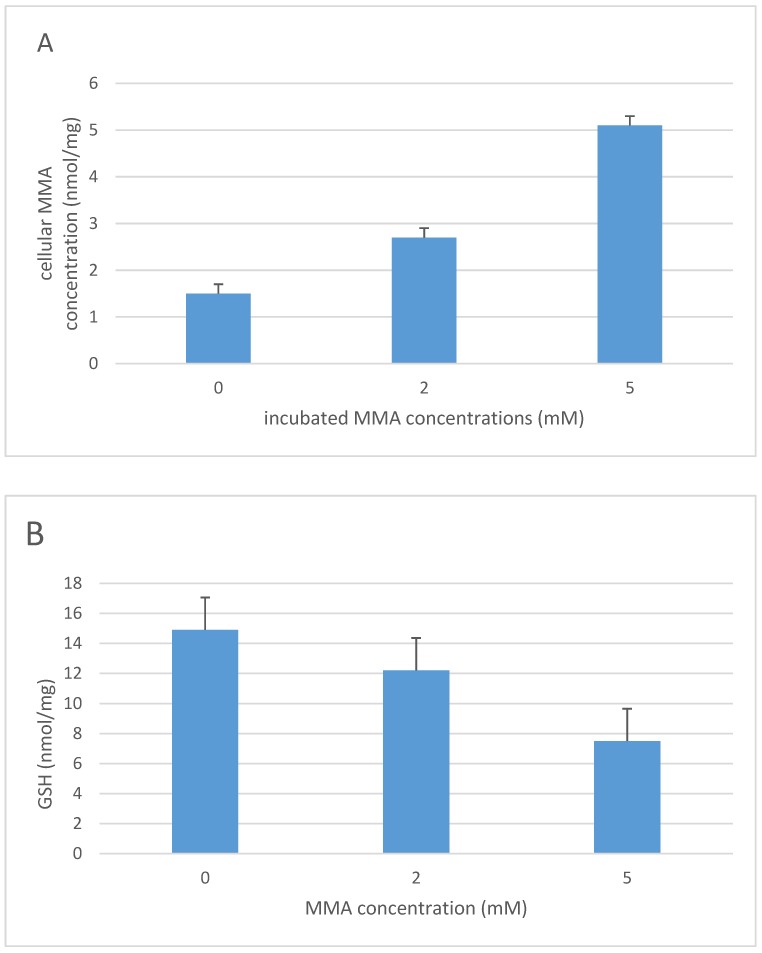
The concentration of cellular MMA (**A**) and GSH (**B**) in human neuroblastoma SHS-5Y cells following 10 days of incubation with MMA (0, 2 and 5 mM). Results are expressed as the mean and standard deviation of five determinations. MMA: Methylmalonic acid; GSH: Reduced glutathione. Previously unpublished data obtained by the authors of this paper with permission given for its publication.

An increase in lactate concentration together with a reduction in *n*-acetyl aspartate were observed in the globus pallidus of patients with methylmalonic acidemia which in conjunction with an elevation in cerebrospinal fluid (CSF) lactate levels indicated a possible perturbation in mitochondrial oxidative metabolism [77]. The pathological changes in methylmalonic acidemia are thought to result from the accumulation of toxic organic acids during decompensation [78], and this toxicity has been ascribed to MMA and its metabolites, methylcitrate and malonate [10,79,80]. However, it has been suggested that the mitochondrial dysfunction observed in methylmalonic acidemia is the result of inhibition of the ETC by methylcitrate and malonate rather than by MMA, which has been reported not to inhibit ETC enzyme activity [10]. Although, results from other studies have suggested the propensity for MMA to inhibit ETC activity [66,68,79,81,82,83,84]. The ETC dysfunction associated with methylmalonic acidemia may therefore be the result of synergistic inhibition of the ETC by MMA, methylcitrate and malonate [59]. Evidence of oxidative stress in methylmalonic acidemia has been reported in a number of studies both in patients [56,85] and animal models [68,86,87,88]. ETC dysfunction is thought to be the major cause of oxidative stress in methylmalonic acidemia [86]; however, increased expression of the mitochondrial enzyme, glycerophosphate dehydrogenase, may also contribute to the ROS generation in this condition [63]. The effect of methylmalonic acidemia on cellular antioxidant status has been documented in a number of studies. In 1996, Treacy et al. reported a blood GSH deficiency in a seven-year-old child with this condition [59]. The patient was treated with high-dose ascorbic acid therapy and showed some clinical improvement which the authors suggested may have resulted as a consequence of the vitamin supplementation eliciting a replenishment of cellular antioxidant capacity. Evidence of a decrease in GSH status was also reported in the liver of a mouse model of methylmalonic acidemia [69]. In this study, a decrease in the level of GSSG (the oxidised form of GSH) was also reported, indicating that an impairment in cellular ATP generation may also have contributed to the loss of total glutathione (GSH + GSSG) status. Since glutathione synthesis is ATP-dependent [89], the ETC deficiencies also reported in the liver tissue of the animal model may have been sufficient to compromise oxidative phosphorylation [69]. Decreased plasma [90] and monocyte levels of GSH [85] have also been reported in patients with methylmalonic acidemia, which in both studies accompanied evidence of increased oxidative stress. In view of the number of toxic organic acids which have been implicated in the pathogenesis of methylmalonic acidemia [10,79,80], the authors investigated the propensity of MMA to induce a deficit in the level of neuronal cell GSH status. In this human neuroblastoma, SHS-5Y cells were incubated with MMA at concentrations reported in the plasma of patients with methylmalonic acidemia (2 and 5 mmol/L) [69]. Cellular GSH levels were determined by the HPLC electrochemical method outlined in the study by Hargreaves et al. (2005) following 6 and 10 days in culture, respectively (Figure 3) [89]. Although no evidence of decrease of GSH status was detected after 6 days of culture, evidence of a significant (*p* < 0.05) decrease in SHS-5Y cell GSH status following 10 days of culture with 5 mmol/L MMA was determined following Student’s *t* test analysis of the data.

**Figure 4:**
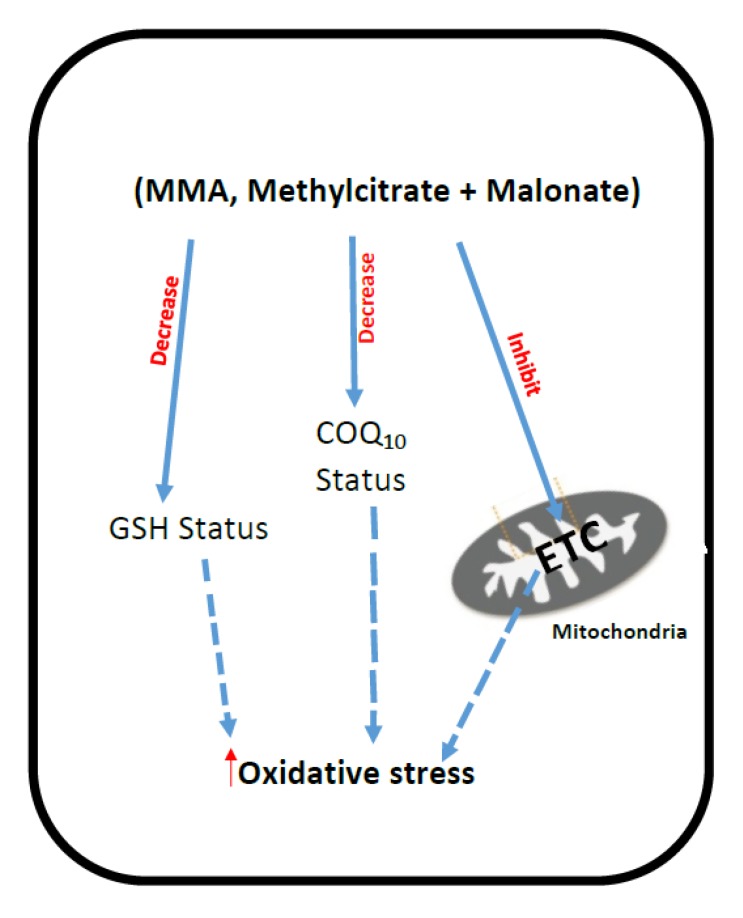
Putative mechanisms of oxidative stress generation and mitochondrial dysfunction in Methylmalonic acidemia. MMA: Methylmalonic acid; ETC: Mitochondrial electron transport chain; CoQ_10_: Coenzyme Q_10_; GSH: Reduced glutathione; ROS: Reactive oxygen species.

The CoQ_10_ status of fibroblasts from patients with MMA as the result of either l-methylmalonyl-CoA mutase deficiency or by defects in the synthesis of 5-deoxyadenosyl cobalamin were found to be significantly (*p* < 0.05) decreased compared with aged-matched controls [91]. Furthermore, a decreased level of CoQ_10_ was also reported in a mouse model of this condition [88]. However, the level of Coenzyme Q_9_, which is the predominant ubiquinone species in mice [92], was comparable to control levels discounting the possibility of impairment in ubiquinone biosynthesis [92]. The putative mechanisms that have been implicated in ETC dysfunction and oxidative stress in methylmalonic acidemia are outlined in Figure 4.

Antioxidant have been recommended as an adjunct therapy to treatment regime of methylmalonic acidemia patients; however, few studies have evaluated their potential therapeutic efficacy [93]. CoQ_10_ treatment in conjunction with vitamin E was reported to improve visual acuity in a 15-year-old methylmalonic acidemia patient with optic neuropathy [94]. Although this report contrasts with a previous case study by Williams et al. (2009), which failed to demonstrate any evidence of visual improvement following CoQ_10_ therapy, vitamin E was not included in the treatment regime of the latter patient [95]. A significant improvement in glomerular filtration rate was also reported in a mouse model of methylmalonic acidemia following co-treatment with CoQ_10_ and vitamin E, suggesting that the beneficial effects of this therapy may not be restricted to the nervous system [88]. In light of evidence demonstrating a deficit in GSH status in methylmalonic acidemia [69,85,90], therapeutic strategies aimed at replenishing this tripeptide may prove beneficial to patients with this condition, although as far as the authors are aware, no such studies have been undertaken.

## 4. Peroxisomal Disorders

Peroxisome disorders are a heterogeneous group of rare metabolic diseases that can result from either a single peroxisomal enzyme deficiency (Refsum disease and X-linked adrenoleucodystrophy; X-ALD) [96] or as the result of a perturbation in the biogenesis of the organelle (Zellweger Syndrome spectrum disorders and rhizomelic chondrodysplasia punctate: RCDP) [97].

Zellweger Syndrome, neonatal adrenoleucodystrophy (ALD) and infantile Refsum disease all belong to the Zellweger spectrum of peroxisome biogenesis disorders and result from mutations in the PEX genes which encode superperoxins, proteins required for the import of protein into peroxisome, as well as the assembly of the organelle [97]. Patients with Zellweger Syndrome spectrum disorders lack functional peroxisomes and, as a result, have matrix proteins from the organelle mislocalized in the cytosol [97].

The disparity in the biochemical and clinical phenotypes of patients with Zellweger Syndrome spectrum peroxisomal disorders suggests that a large set of PEX mutations is likely to contribute to their pathogenesis, possibly via additional molecular mechanisms independent of their role in peroxisome biogenesis [98]. Clinical manifestation of Zellweger Syndrome group of disorders varies and includes liver disease, variable neurodevelopmental delay, retinopathy and sensorineural deafness. Patients with RCDP disorders present with skeletal dysplasia including proximal shortening of the limbs (rhizomelia) and punctate calcifications in cartilage present at birth, profound growth deficiency, cataracts and severe psychomotor defects [99]. ALD is the most frequent inherited leukodystrophy and peroxisomal disorder, characterized by an inflammatory cerebral demyelination, or a progressive axonopathy in the spinal cord, causing spastic paraparesis [100,101,102].

Peroxisomes have multiple biosynthetic functions and play a role in the β-oxidation of very-long-chain fatty acids (VLCFA) [103], prostaglandins, dicarboxylic acids, xenobiotic fatty acids and hydroxylated 5-β-cholestanoic acids [104]. In peroxisomal β-oxidation, the electrons liberated during the degradation of very-long-chain acyl-CoAs (VLCAC) are transferred directly to oxygen to generate hydrogen peroxide (H_2_O_2_) [105]. In addition, peroxisomes also contain a number of other ROS-generating enzymes such as Xanthine oxidase, which liberates H_2_O_2_ and superoxide during the catabolism of purines, and therefore these organelles are a major site of ROS generation within the cell [106]. In order to compensate for the abundance of ROS generated, the peroxisomes are well equipped with antioxidant defense systems, most notable of these being the catalase enzyme which converts H_2_O_2_ to oxygen and water [107]. Therefore, not unsurprisingly, peroxisomal disorders have been associated with oxidative stress, which is thought to contribute to disease progression [108,109]. The origin of oxidative stress in this disorder is thought to result from either an impairment of the peroxisomal antioxidant defense system and/or an accumulation of VLCFAs as well as VLCACs from the β-oxidation system of this organelle [110]. Peroxisomes also contain the inducible form of NOS, iNOS which catalyses the oxidation of *L*-arginine to citrulline and NO [111]. However, in peroxisomes this enzyme is thought to exist in its inactive monomeric form, whilst the cytosol contains both the monomeric and active homodimer forms of the enzyme [111]. Although, it has been speculated that under the circumstances peroxisomal iNOS may produce NO and this may be an explanation for the significant (*p* = 0.022) increase in NO*x* (marker of NO production) reported in the serum of patients with peroxisomal biogenesis disorders in the study by El-bassyouni et al. (2012) [109].

Peroxisome biogenesis defects resulting from PEX gene mutations may impair the import of matrix proteins such as catalase [112], impairing the antioxidant capacity of the organelle and rendering the cell more susceptible to free radical-induced oxidative damage [113]. This is also observed in aging cells where catalase is also mislocalized to the cytosol, resulting in an accumulation of cellular ROS with associated damage to protein, lipids and DNA [114]. In addition, peroxisomal biogenesis defects will also cause an impairment in the synthesis of the phospholipid antioxidant species, plasmalogens, which will compromise the ability of the cell to detoxify ROS [115,116].

**Figure 5:**
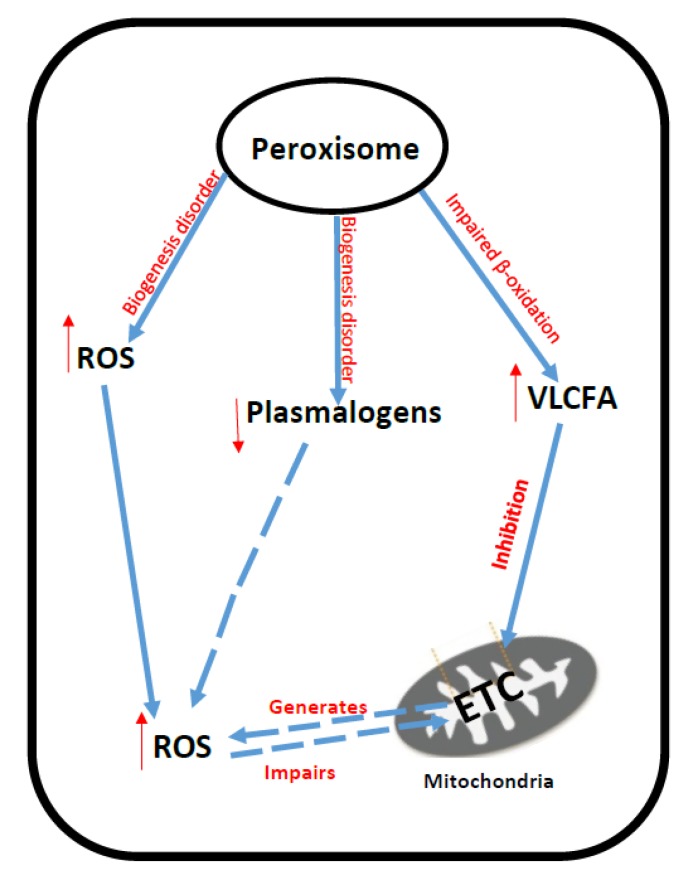
Putative mechanisms of oxidative stress generation and mitochondrial dysfunction in peroxisome disorders. VLCFA: Very-long-chain fatty acid; ROS: Reactive oxygen species; ETC: Mitochondrial electron transport chain.

Studies in fibroblasts from patients with X-ALD have revealed that hexacosanoic acid (C26:0), the VLCFA which accumulates in this disorder, causes a direct impairment of oxidative phosphorylation resulting in an increase in ROS generation and, consequently, the oxidation of mitochondrial DNA and proteins [117]. The mechanism by which C26:0 impairs oxidative phosphorylation in X-ALD is as yet uncertain, but may result from the ability of C26:0 to disrupt the physicochemical properties of the mitochondrial membrane [118]. The accumulated VLCFAs and VLCACs resulting from peroxisomal dysfunction may directly impair ETC function causing an increase in ROS generation from the chain as illustrated by the ability of phytanic acid, the C20 branch fatty acid that accumulates in Refsum disease to inhibit ETC complex I activity whilst concomitantly causing mitochondrial oxidative stress [11]. Since a number of studies have reported evidence of impaired oxidative phosphorylation in peroxisomal disorders [108,119,120,121,122,123,124], and the ETC is a major source of ROS generation [13], it does appear judicious to suggest that mitochondrial dysfunction may be a major contributor to the oxidative stress detected in these diseases [125,126,127]. The putative mechanisms that have been implicated in ETC dysfunction and oxidative stress in peroxisomal disorders are outlined in Figure 5.

In an animal model of X-ALD, oxidative damage, metabolic failure and axonal degeneration were reversed following treatment with the antioxidants, *n*-acetyl cysteine (NAC), α-lipoic acid, and α-tocopherol, providing proof of concept on the pivotal contribution of oxidative damage to disease pathogenesis in addition to illustrating the efficacy of antioxidant interventions [128,129,130]. A subsequent human study in X-ALD documented the ability of NAC treatment to replenish plasma GSH levels and ameliorate oxidative damage to proteins under in vitro conditions [131]. Evidence of decreased plasma CoQ_10_ status was reported in patients with a defect in peroxisome β-oxidation enzyme, d-bifunctional protein, which was associated with markers of increased oxidative stress [132]. It has been suggested that, based on the integral involvement of oxidative stress in the pathogenesis of peroxisomal disorders, the administration of antioxidants should be considered as a potential adjunct therapy for patients with these diseases [109,131,132].

## 5. Xeroderma Pigmentosum

Xeroderma pigmentosum (XP) is a rare condition characterized by an extreme sensitivity to ultraviolet (UV) rays from sunlight often causing skin burn. It affects the eyes and areas of skin exposed to the sun and is associated with an increased risk of skin cancer of lips, eyelids as well as brain tumors [133]. Patients with XP may present with neurological complications such as cerebellar ataxia, chorea, hearing loss, poor coordination, difficulty walking, movement problems, loss of intellectual function, difficulty swallowing and talking, and seizures [134]. Mutations in eight genes have been associated with XP.

XP is caused by autosomal recessive mutations in genes encoding for proteins that play a role in the nucleotide excision repair system (NER) [135]. There are eight complementation groups of XP, seven correspond to dysfunctional NER complex components, XP-A to -G, and one which affects DNA polymerase-η involved in translation synthesis and post-replication repair: XP-Variant (XP-V) [136]. XP cells lack a functional NER mechanism and so UV-induced bulky DNA lesions resulting from exposure to UV rays cannot be corrected. Unrepaired lesions occur in many genes, including those that encode cell growth and proliferation factors leading to a high rate of mutagenesis during DNA replication [137]. As well as the role of NER in UV-induced DNA damage repair, there is increasing support for the involvement of NER proteins in the repair of oxidative DNA damage [138,139]. Evidence of oxidative DNA damage in the form of free radical-induced DNA lesions such as 8-hydroxy-2-deoxygyanosine and cyclodeoxypurines have been detected in tumours and autopsied brains of neurological XP patients and animal models [140,141,142]. In XP-A, no evidence of DNA repair was reported in a study by Hayahi et al. (2008) and lesions were found to accumulate in patient cells [143]. The accumulation of such unrepaired DNA may be the source of internal carcinogenesis [144] and neuronal cell death, explaining the progressive neurodegeneration in XP [139,142].

Studies have been undertaken to elucidate the origin of oxidative stress in XP-C, the commonest form of this condition in Caucasians [145], and have indicated that the activation of the cytosolic enzyme, NADPH oxidase (NO*x*), may be a major contributor to ROS generation in this disease [146,147]. Furthermore, the NO*x* activation-induced ROS production has been suggested as a possible cause of the mitochondrial dysfunction detected in XP-C and possibly other forms of XP [146]. However, a study by Fang et al. (2014) suggested that impaired mitophagy may also contribute to the mitochondrial dysfunction observed in XP-A [148]. Interestingly, impaired mitophagy has also been associated with increased cellular ROS generation [149].

Evidence of mitochondrial dysfunction in XP has been indicated by mitochondrial DNA (mtDNA) deletions [150,151], ETC enzyme dysfunction [147,152] and morphological abnormalities [153,154]. Interestingly, studies have suggested that mitochondria are the major source of ROS generation in human XP-C cells and that mtDNA is the primary target for damage accumulation [152]. Since mtDNA lacks an NER, with repair being elicited through other mechanisms [155], this does suggest that mitochondrial abnormalities reported in XP are a secondary consequence of abnormalities in the nuclear DNA repair system.

**Figure 6:**
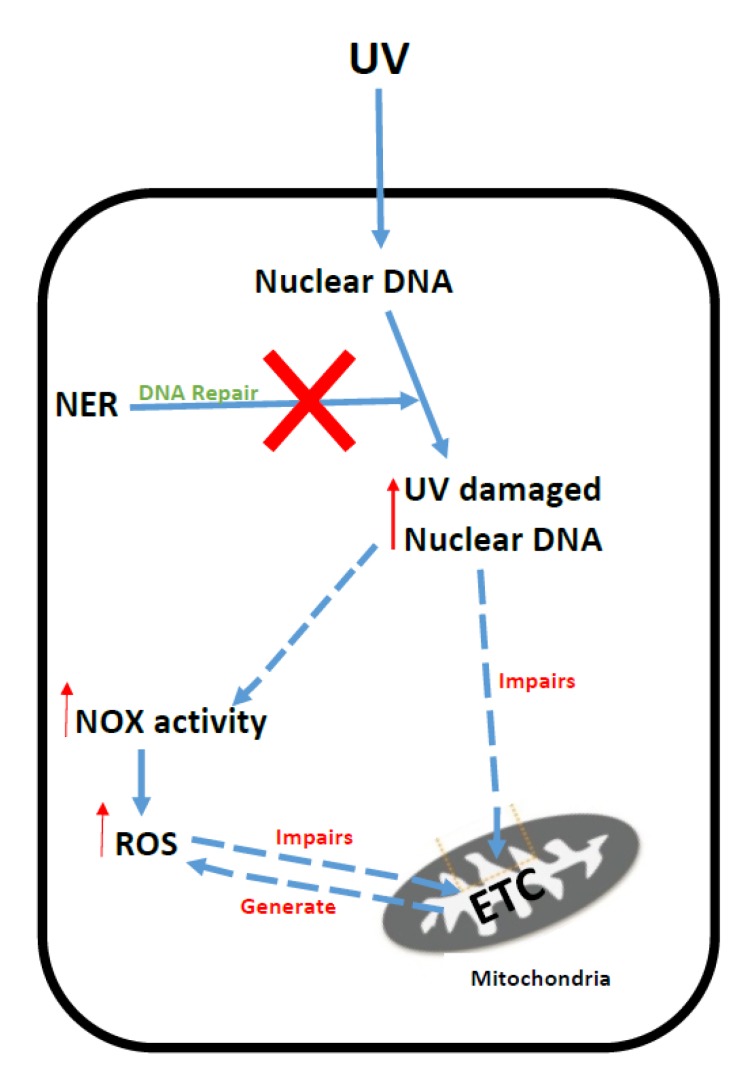
Putative mechanisms of oxidative stress generation and mitochondrial dysfunction in Xeroderma Pigmentation. UV: Ultraviolet radiation; NER: Nucleotide excision repair system; NO*x*: NADPH oxidase; ETC: Mitochondrial electron transport chain; ROS: Reactive oxygen species.

Decreased activities of the antioxidant enzymes, catalase [156], SOD (superoxide dismutase) [143] and GSH-PX [152] have been reported in patient tissue and cell models of XP. In addition, decreased plasma CoQ_10_ levels were reported in patients with XP, with improvements in their daily activity being documented in a subset of these patients following CoQ_10_ supplementation [157]. The putative mechanisms that have been implicated in ETC dysfunction and oxidative stress in XP are outlined in Figure 6.

The authors are aware of no studies as yet to evaluate the therapeutic potential of antioxidants in the treatment of XP, although genetic strategies to ameliorate ROS generation are being considered [158].

## 6. Sepsis

Sepsis is a chain of pathophysiological and metabolic reactions in response to infection, also identified as the systemic inflammatory response syndrome (SIRS) [16,159]. Clinically, sepsis may present in different forms depending on severity and include SIRS, septic shock and, in severe cases, multiple organ dysfunction syndrome including septic shock. The mortality rate is significantly increased (up to 34%) in patients with acute kidney injury versus 7% in patients without acute kidney injury [160]. Sepsis, together with hypoperfusion, is responsible for half of all cases of acute kidney injury in Intensive Care Units [161,162,163].

The precise pathophysiologic mechanisms underlying the development of multi-organ failure remain elusive [164]. However, the main causes of sepsis have been identified and include infection by gram-positive and gram-negative bacteria, fungi, or both. Concomitant factors, such as diabetes, transplantation, surgical intervention, chronic obstructive pulmonary disease, congestive heart failure, and renal disease increase a person’s susceptibility to sepsis or aggravate their clinical score [16]. Additionally, an excessive degree of inflammation in response to the infectious insult triggers an activation of multiple downstream pathways. As a result, activated leukocytes release inflammatory cytokines such as tumour necrosis factor (TNF)-a, IL-1a, IL-1b, and IL-6, and chemokines such as IL-8 and KC that also impact upon the severity of sepsis [16]. Sepsis-related organ failure is associated with a significant morbidity and mortality [165,166] with long-term physical and neurocognitive problems affecting many survivors of critical illness [167,168].

It has been suggested for many years that both oxidative and nitrosative stress play a central role in the pathogenesis of sepsis and that ETC dysfunction may be an important causative factor in the multi-organ dysfunction associated with this condition [16]. Within the confines of this review, it would not be possible to outline all the mechanisms that have been proposed to account for the generation of free radical species or ETC dysfunction reported in sepsis. However, a paradigm will be offered based on the results of studies from the literature.

The inflammatory cytokines released by activated leukocytes following exposure to exo- and endo-toxins (most notably lipopolysaccharides; LPS) produced by gram-positive and -negative bacteria, respectively cause the overproduction of the RNS, NO, by the induction of iNOS activity in a number of vital organs including the heart and kidney as well as skeletal muscle [169,170,171]. LPS treatment has also been reported to induce NO*x* expression in renal cells resulting in a concomitant increase in ROS production [172].

**Figure 7:**
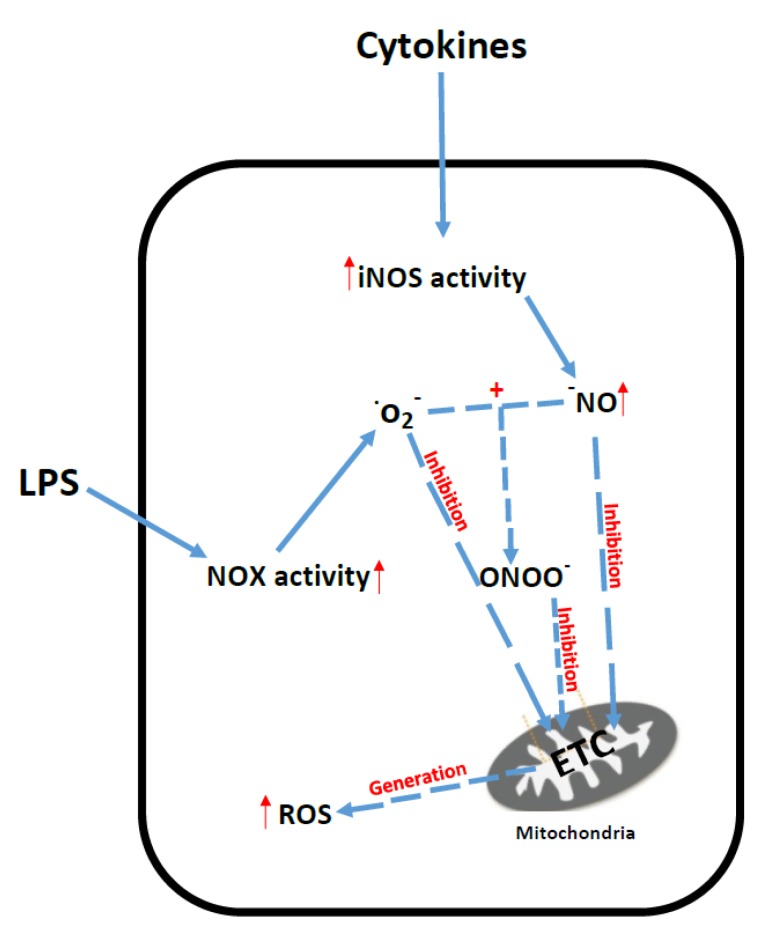
Putative mechanisms of oxidative stress and mitochondrial dysfunction in sepsis. iNos: Inducible nitric oxide synthase; NO*x*: NADPH oxidase; O_2_^−^: Superoxide; NO: Nitric oxide; ROS: Reactive oxygen species; ETC: Mitochondrial electron transport chain; ONOO^−^: Peroxynitrite; LPS: Lipopolysaccharides.

The over-production of ROS and RNS by the cell may then result in the impairment of ETC function [2,12]. NO can combine with the ROS species, superoxide, to form the highly RNS species peroxynitrite, which can cause irreversible inhibition of the ETC [173]. Multiple ETC enzyme deficiencies have been reported in patients and animal models of sepsis [174,175]. As a consequence of ETC dysfunction, the mitochondria may also become a source of cellular ROS generation in sepsis, which can further exacerbate oxidative phosphorylation [172]. Decreased tissue ATP levels associated with ETC dysfunction have been linked to both organ failure and an increased mortality rate in sepsis [2]. The putative mechanisms that have been implicated in ETC dysfunction and oxidative stress in sepsis are outlined in Figure 7.

In view of the ability of the ROS and RNS generated in sepsis to overwhelm cellular antioxidant defenses [2] and inhibit ETC function, a number of therapeutic strategies aimed at replenishing cellular antioxidant status have been investigated in patients and animal models of the disease [16]. The ability to replenish tissue GSH levels which have been found to be deficient in sepsis patients has been associated with clinical and biochemical improvement in animal models [176,177]. In addition, Se supplementation has been associated with increased GSH-PX activity [178] and a decreased mortality rate in septic patients [179]. It has been suggested, however, that mitochondrial-targeted antioxidants using compounds such as MitoQ or mitoVit E may offer novel therapeutic avenues to explore in the future [180]. Although, ubiquinol, the reduced form of CoQ_10_ has been reported to reduce peroxynitrite levels and attenuate the damage of the ETC associated with this RNS [181].

## 7. Conclusions

Mitochondrial dysfunction and oxidative stress are inextricably linked to the pathophysiology of a number of diseases as indicated by the disorders referred to in this review (Table S1). Within the mitochondria, the ETC is particularly vulnerable to ROS- and RNS-induced impairment either as the result of oxidative damage to the enzyme complexes, mtDNA or the mitochondrial membrane phospholipids [182,183]. Once impaired, the ETC then becomes a major source of ROS generation, resulting in further ETC dysfunction and compounding cellular oxidative stress [13,184]. The cell possesses a number of antioxidant defense systems to combat ROS and RNS; however, during pathological condition these defenses become overwhelmed, causing oxidative damage to the biomolecules of the cell and resulting in cellular and, consequently, organ dysfunction [9]. The use of appropriate antioxidants as an adjunct therapy may be particularly important in the treatment of diseases associated with oxidative stress, although treatment protocols have yet to be standardized or indeed instigated in some clinical centres. Since the mitochondria can make a major contribution to cellular oxidative stress in the disease state, antioxidant strategies which target this organelle may offer particular therapeutic potential [180]. Evidence of oxidative stress can be detected in patients through non-invasive means such as by assessing plasma antioxidant status or the stable end products of lipid, DNA or protein oxidation as alluded to in this review. For this reason, it is particularly important to engender some consensus with an aim to establishing a unified approach to monitoring evidence of oxidative stress in patients with diseases associated with this parameter together with the development of appropriate therapeutic strategies. It is also essential to take into account the possibility of nitrosative as well as oxidative stress in patients, the former being implicated as a major contributory factor to a number of chronic diseases and conditions [185]. In diseases which have been associated with oxidative stress and/or nitrosative stress, it is important to firstly, select a reliable and sensitive marker of this/these parameter(s) and, secondly, to choose an appropriate surrogate tissue for monitoring purposes. In the clinical studies outlined in this review, a number of different end-point markers were used to monitor evidence of oxidative stress and the sensitivity and specificity of these markers may vary [31,33,34,108,109]. In addition, in view of the sophistication and/or laboriousness of a number of these methods, it may be difficult to translate them into a clinical laboratory setting. Therefore, the ability to assess a number of markers of both oxidative and nitrosative stress together in a large-scale panel by either Liquid chromatography-mass spectrometry and/or ELISA as suggested by Frijhoff et al. (2015) [186] may overcome the problems of sensitivity/specificity as well as decrease the assay time for these determinations. The surrogate which is generally used to measure levels of ROS, RNS as well as antioxidant status in clinical studies [33,34,132,178,179] is plasma/serum; however, it is uncertain whether levels of these parameters in plasma/serum reflect those of tissue. This is certainly the case for CoQ_10_, and plasma CoQ_10_ status has been reported not to reflect that of muscle [187]. Blood mononuclear cells or lymphocytes have been suggested as appropriate alternative surrogates for this determination in patients [43,44]. Furthermore, lymphocytes have also been suggested as an appropriate surrogate to assess intracellular GSH status in patients [188]. Therefore, the assessment of ROS, RNS or antioxidant status in white blood cells rather than plasma/serum in future clinical studies may give a better indicator of these parameters in tissue. A compound to consider for future treatment strategies in diseases associated with mitochondrial dysfunction and oxidative and nitrosative stress is the synthetic quinone, EPI-743 [188]. This compound has demonstrated some therapeutic efficacy in the treatment of patients with primary mitochondrial disorders by its ability to replenish cellular GSH status as well as its proposed capacity to interact with the transcription factor, nuclear factor E2-related factor 2 (Nrf2) which regulates both the expression of antioxidant proteins as well as cellular energy metabolism [189,190]. However, one reason why antioxidants in general have been relatively ineffective in treating either acute or chronic diseases is that they are only targeting oxidative stress and do not take into account nitrosative stress, which can make a major contribution to disease pathophysiology in a number of disorders [2,12]. Therefore, antioxidant treatments that target both oxidative as well as nitrosative stress are important considerations for future therapeutic strategies.

## Supplementary Materials

Supplementary File 1

## Abbreviations

ALD	adrenoleuklodystrophy
CSF	cerebrospinal fluid
ETC	electron transport chain
CoQ_10_	coenzyme C_10_
Fe	iron
GSH	glutathione
GSH-PX	glutathione peroxidase
GSSG	the oxidised form of GSH
HMG-CoA	3-hydroxy-3-methylglutaryl-CoA
H_2_O_2 _	hydrogen peroxide
LPS	lipopolysaccharides
Inos	induction of nitric oxide synthase
MCM	l-methylmalonyl-CoA mutase
MMA	methylmalonic acid
mtDNA	mitochondrial DNA
NADPH	nicotinamide adenine dinucleotide phosphate-oxidase
NAC	*N*-acetyl-cysteine cysteine
NER	nucleotide excision repair system
NO*x*	NADPH oxidase
Phe	phenylalanine
PKU	Phenyloketonuria
RCDP	rhizomeric chondrodysplasia punctate
SOD	superoxide dismutase
SIRS	systemic inflammatory response syndrome
ROS	reactive oxygen species
RNS	reactive nitrogen species
Se	selenium
VLCFA	very-long-chain fatty acids
VLCAC	very-long-chain acyl-CoAs
TBAR	thiobarbituric acid-reactive species
XP	Xeroderma pigmentosum
