# Supplementary material for: Evidence of Oxidative Stress and Secondary Mitochondrial Dysfunction in Metabolic and Non-Metabolic Disorders
Source: J Clin Med. 2017 Jul 19;6(7):71. doi: 10.3390/jcm6070071 (PMC5532579; doi:10.3390/jcm6070071)
Supplement: Supplementary File 1 [file jcm-06-00071-s001.docx]

**Table 1. References in this review reporting evidence of mitochondrial dysfunction, oxidative and nitrosative stress in phenyloketonuria, methylmalonic acidemia, peroxisomal disorders, xeroderma pigmentosum and Sepsis.**

| **Disease** | **References indicating evidence of mitochondrial dysfunction** | **References indicating evidence of oxidative stress** | **References indicating evidence of nitrosative stress** |
| --- | --- | --- | --- |
| **Phenyloketonuria** | 43,44,45,46,47 | 15,31,32,33,34,35,37,38,39 | 33 |
| **Methylmalonic acidemia** | 10, 64, 65,67,68,69,70,71,72,73,74, 79,81,83,84,85,86 | 65,70, 87, 88, 89, 90, 91,94 |  |
| **Peroxisomal disorders** | 119,121,122,123,124,125,126,127,128 | 111,112,119,121,129,130,131,132,133, 136 | 112 |
| **Xeroderma pigmentosum** | 151,152, 154, 155,156, 157, 158,161 | 142, 143,147,150,151,155 |  |
| **Sepsis** | 16, 176,177, 178, 179, 180, 181, | 16,176,177,178,186 | 16,173,174,175,177,178 |

Numbers represent references used in this review.
